# Effects of Intensive Systolic Blood Pressure Control on Glycometabolic and Cardiovascular Outcomes in Normoglycemic Patients: A Secondary Analysis of a Randomized Trial

**DOI:** 10.1002/mco2.70197

**Published:** 2025-05-07

**Authors:** Cheng Yang, Wei‐Hua Chen, Jie Qian, Rong‐Chong Huang, Jian‐Jun Li

**Affiliations:** ^1^ Cardiometabolic Center, Fuwai Hospital, National Center for Cardiovascular Diseases, Chinese Academy of Medical Sciences and Peking Union Medical College Beijing China; ^2^ Department of Cardiology Beijing Friendship Hospital, Capital Medical University Beijing China

1

Dear Editor,

The increasing prevalence of diabetes mellitus is a challenge to the global healthcare landscape, imposing a substantial burden of complications and contributing to premature mortality [1]. Prediabetes, an intermediate stage between normoglycemia and diabetes, is a well‐established risk factor for progression to overt diabetes. Prediabetes and diabetes (collectively termed dysglycemia) are direct and independent risk factors for cardiovascular disease (CVD) [2]. Hypertension and diabetes commonly overlap in the population, exacerbating increased cardiovascular morbidity and mortality [3]. The relationship between blood pressure (BP) control and dysglycemia is not yet fully elucidated. Posthoc analyses of two major randomized controlled trials, the Systolic Blood Pressure Intervention Trial (SPRINT) and the Strategy of Blood Pressure Intervention in the Elderly Hypertensive Patients (STEP), have yielded contradictory conclusions regarding the impact of BP intervention on glucose metabolism [4, 5]. The risk of impaired glucose tolerance associated with intensive systolic BP lowering raises concerns about its benefits. Indeed, abnormal glucose metabolism increases additional cardiovascular risk compared with hypertensive patients exhibiting normal blood glucose levels. There is still a lack of detailed studies on the complex relationship between intensive BP control and the development of abnormal glucose metabolism. Furthermore, the cardiovascular risk in individuals who develop abnormal glucose metabolism as a result of intensive BP lowering remains insufficiently examined. Comprehensively understanding the risks and benefits associated with aggressive BP management is essential for informed decision‐making by both patients and healthcare providers. We aimed to provide novel insights into the association between intensive BP control and glycometabolic outcomes within the SPRINT population, as well as to evaluate the subsequent cardiovascular outcomes.

Among the 9361 SPRINT participants, those with missing baseline blood glucose data (*N* = 618), baseline blood glucose levels ≥100 mg/dL (5.6 mmol/L) (*N* = 3657), or a history of diabetes or use of antidiabetic medications at baseline (*N* = 179) were excluded. Finally, 5027 participants with normoglycemia at baseline were included in the current analysis (2522 from the intensive BP lowering and 2505 from the standard BP lowering; Figure 1A). Over a median follow‐up of 3.76 years, a total of 823 (37.7%) subjects developed dysglycemia, 242 (9.6%) developed a composite of CVD event or all‐cause death, 162 (6.4%) developed CVD event, and 121 (4.9%) deaths occurred in the intensive treatment group. In the standard group, a total of 701 (32.3%) subjects developed dysglycemia, 300 (12.0%) developed a composite of CVD event or all‐cause death, 209 (8.3%) developed CVD event, and 152 (6.1%) deaths occurred. These results indicated a higher incidence of dysglycemia in the intensive group compared with the standard group.

**FIGURE 1:**
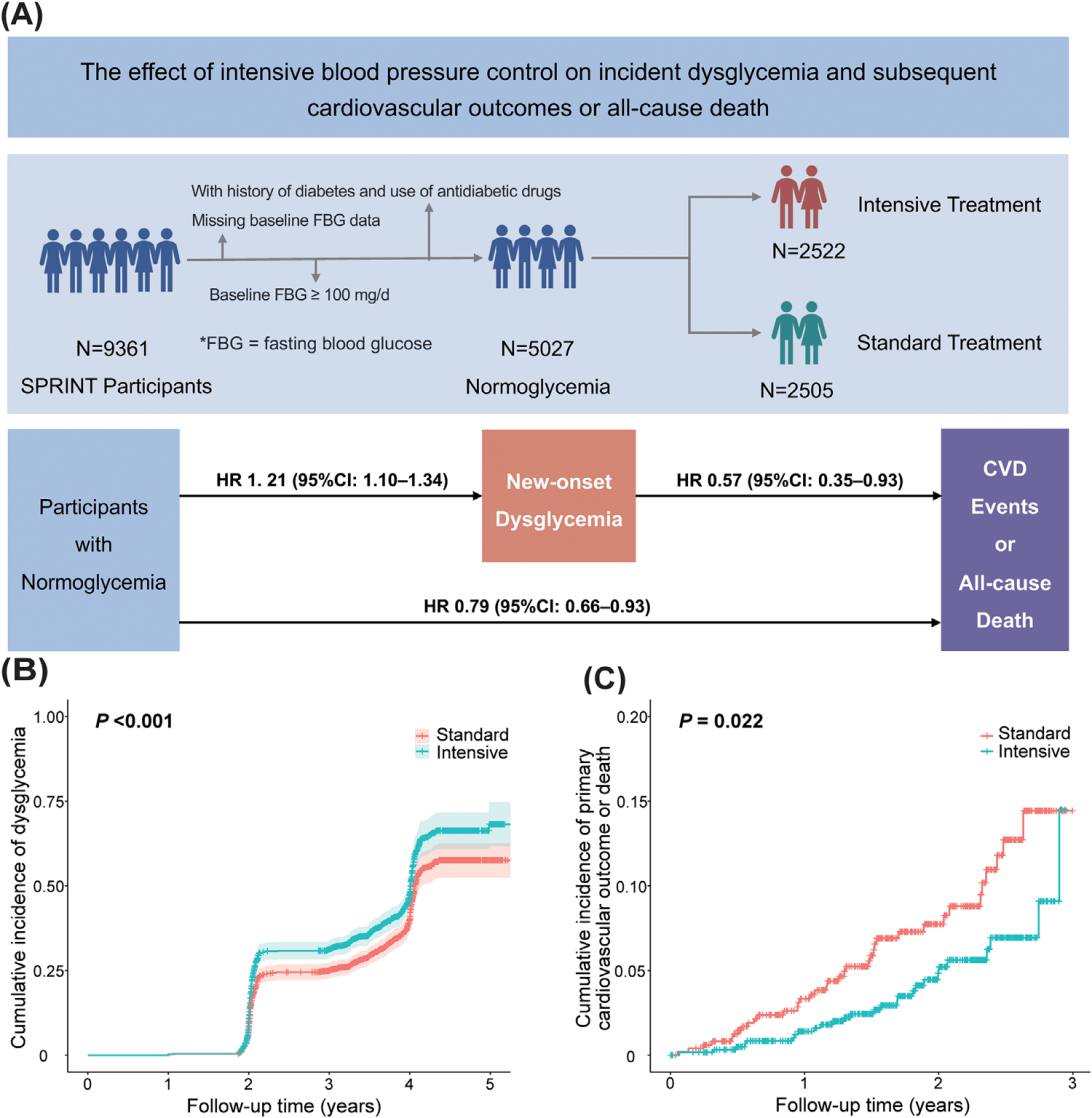
Impact of intensive blood pressure control on dysglycemia incidence and subsequent cardiovascular outcomes or all‐cause death. (A) Study population flow diagram. Among 9361 participants from the Systolic Blood Pressure Intervention Trial (SPRINT), we excluded those with baseline dysglycemia (fasting glucose ≥100 mg/dL), diabetes history, use of antidiabetic medications, or missing glucose data at baseline. The final analysis included 5027 normoglycemic individuals randomized to intensive (*n* = 2522) or standard (*n* = 2505) blood pressure lowering. Among participants with normoglycemia, intensive blood pressure lowering was associated with a higher risk of incident dysglycemia (HR 1.21, 95% CI 1.10–1.34), but this was offset by the benefits of reduced cardiovascular events and all‐cause mortality (HR 0.79, 95% CI 0.66–0.93). (B) Cumulative incidence of dysglycemia in participants with normoglycemia. (C) Kaplan–Meier curves of cardiovascular outcome or all‐cause death in patients with new‐onset dysglycemia. HR, hazard ratio; CI, confidence interval; CVD, cardiovascular disease.

For participants with normoglycemia at baseline, compared with the standard group, the intensive group had a significantly higher rate of incident dysglycemia (114 vs. 96.2 events per 1000 person‐years; HR 1.21, 95% confidence interval [CI]: 1.10–1.34, *p* < 0.001; Figure 1A, B). At 3 years, the cumulative incidence of dysglycemia was an absolute 3.9% (95%CI: 3.8–4.0%) higher in the intensive group. Subgroup analyses of SPRINT participants stratified by age, sex, and race showed consistent results across all subgroups, with no significant differences observed. The intensive BP control significantly reduced the risk for the composite of a primary CVD event or all‐cause death (HR 0.79, 95%CI: 0.66–0.93, *p* = 0.008), primary CVD event (HR 0.76, 95%CI: 0.62–0.93, *p* = 0.006), and all‐cause death (HR 0.79, 95%CI: 0.62–1.00, *p* = 0.047). For subjects with new‐onset dysglycemia, the intensive BP control significantly reduced the risk for the composite of a CVD outcome or all‐cause death (HR 0.57, 95%CI: 0.35–0.93, *p* = 0.024; Figure 1A,C), CVD event (HR 0.56, 95%CI: 0.31–1.01, *p *= 0.053), and all‐cause death (HR 0.42, 95%CI: 0.21–0.83, *p* = 0.012). The findings were consistent across subgroups of SPRINT participants stratified by age, sex, race, and statin use. Therefore, among SPRINT participants with normoglycemia, intensive BP lowering, compared with standard BP lowering, resulted in both an absolute and relative increase in the risk of incident dysglycemia. However, this risk was offset by the benefits of reduced CVD outcomes or all‐cause death associated with intensive BP lowering.

Possible mechanisms for the impact of intensified BP intervention on dysglycemia include sympathetic nervous system activation, reduced renal perfusion affecting glucose metabolism, and the metabolic effects of antihypertensive medications, such as diuretics and β‐blockers, which may contribute to insulin resistance.

This study has limitations, including the lack of glycated hemoglobin data and the relatively short follow‐up period, which restricts the inference of the long‐term effects of intensive BP lowering on glycometabolism and cardiovascular outcomes. Therefore, studies involving longer follow‐up time are warranted to investigate these effects.

In conclusion, this secondary analysis of the SPRINT trial, for the first time, demonstrated that while intensive BP lowering increased the risk of new‐onset dysglycemia, this risk was outweighed by the significant benefits in reducing cardiovascular events and all‐cause mortality. Further long‐term studies are warranted to better understand the relationship between intensive BP control and incident dysglycemia.

## Author Contributions

C. Y. contributed to the conception and design of the study, performed data acquisition, conducted statistical analyses, interpreted the data, and drafted the initial version of the manuscript. W. H. C. was involved in the conception and design of the study, contributed to data acquisition, performed statistical analyses, interpreted the results, and revised the manuscript. J. Q. contributed to data interpretation, provided critical insights into the analysis, discussed the results, contributed to shaping the study's strategic direction, and revised the manuscript. R. C. H. supervised the data collection process, provided methodological guidance, contributed to the analysis and interpretation of results, and reviewed and revised the manuscript. J. J. L., the principal investigator of the study, oversaw all aspects of the research, including study design, data interpretation, and result validation. J. J. L. provided critical guidance on analytical strategies, discussed the findings, and contributed to manuscript revisions. All authors reviewed and approved the final version of the manuscript.

## Conflicts of Interest

The authors declare no conflicts of interest.

2

## Ethics Statement

The SPRINT study protocol was approved by the institutional review boards at each clinical site, and all participants provided written informed consent.

## Supporting information




Supporting Information

## Data Availability

SPRINT trial data for this study are publicly available at National Heart, Lung, and Blood Institute BioLINCC data repository.
