# Supplementary material for: Effects of Intensive Systolic Blood Pressure Control on Glycometabolic and Cardiovascular Outcomes in Normoglycemic Patients: A Secondary Analysis of a Randomized Trial
Source: MedComm (2020). 2025 May 7;6(5):e70197. doi: 10.1002/mco2.70197 (PMC12056500; doi:10.1002/mco2.70197)
Supplement: Supplementary file 1 — Supporting Information [file MCO2-6-e70197-s001.docx]

Effects of Intensive Systolic Blood Pressure Control on Glycometabolic and Cardiovascular Outcomes in Normoglycemic Patients: A Secondary Analysis of a Randomized Trial

Authors: Cheng Yang ^1,#^, Wei-Hua Chen ^2,#^, Jie Qian ^1,^*****, Rong-Chong Huang ^2,^*****, Jian-Jun Li ^1,^*****

Affiliations:

^1^ Cardiometabolic Center, Fuwai Hospital, National Center for Cardiovascular Diseases, Chinese Academy of Medical Sciences and Peking Union Medical College, Beijing, 100037, China.

^2^ Department of Cardiology, Beijing Friendship Hospital, Capital Medical University, Beijing, 100053, China.

^#^ Cheng Yang and Wei-Hua Chen contributed equally to this work

^*^Corresponding Authors: Jie Qian, Rong-Chong Huang, and Jian-Jun Li.

Rong-Chong Huang, Department of Cardiology, Beijing Friendship Hospital, Capital Medical University, No. 95 Yong'an Road, Xicheng District, Beijing, 100053, China.

Jie Qian and Jian-Jun Li, Cardiometabolic Center, Fuwai Hospital, National Center for Cardiovascular Diseases, Chinese Academy of Medical Sciences and Peking Union Medical College, No. 167 Beilishi Road, Xicheng District, Beijing 100037, China.

E-mail: [qianjfw@163.com](mailto:qianjfw@163.com) (Jie Qian), [rchuang@ccmu.edu.cn](mailto:rchuang@ccmu.edu.cn) (Rong-Chong Huang), [lijianjun938@126.com](mailto:lijianjun938@126.com) (Jian-Jun Li)

**Materials and Methods**

**Study Population**

SPRINT was a randomized, controlled, open-label trial conducted at 102 clinical sites across the United States and Puerto Rico. Subjects were randomly assigned to the intensive treatment group (target SBP <120 mm Hg) versus the standard treatment group (target SBP <140 mm Hg). The study was approved by the institutional review boards at each clinical site (ClinicalTrials.gov registration no. NCT00145925).

Participants were recruited between November 2010 and March 2013 and were required to meet all of the following inclusion criteria: age ≥50 years, SBP of 130 to 180 mm Hg, and an increased risk of CVD (defined as one or more of the following: clinical or subclinical CVD other than stroke; chronic kidney disease with an estimated eGFR of 20 to less than 60 mL/min/1.73 m^2^; 10-year risk for CVD ≥15%, based on the Framingham global risk score; or aged ≥75 years). Major exclusion criteria included presence of diabetes, prior stroke, fasting proteinuria greater than 1 g/day, polycystic kidney disease, congestive heart failure (symptoms or ejection fraction <35%), dementia, or residence in a nursing home.

For this subgroup analysis of the SPRINT, we further excluded participants who may have initiated the trial with diabetes, were missing fasting blood glucose (FBG) data at randomization, had a FBG of 100 mg/dL (≥5.6 mmol/L) or higher at randomization, or were taking glucose-lowering medication. Baseline characteristics of SPRINT participants with normoglycemia by treatment group are summarized in Table S1.

**Measurement**

The concentrations of FBG were measured at baseline and at follow-up time points of 12, 24, and 48 months or close out visits, utilizing the hexokinase method on a Roche analyzer at the central laboratory. Medical histories were compiled annually, encompassing any reported usage of hypoglycemic medications and documented diagnoses of diabetes. Event ascertainment and safety assessments followed the established protocol guidelines.

**Study Outcomes**

The primary outcome in SPRINT was primary CVD outcomes, defined as a composite of nonfatal myocardial infarction, acute coronary syndrome not resulting in myocardial infarction, non-fatal stroke, non-fatal acute decompensated heart failure, or death from CVD. Secondary outcomes included the components of the primary outcome, all-cause death, and a composite of the primary outcome or all-cause death. All outcome events were adjudicated by an outcomes committee that was blinded to the intervention. A structured interview was conducted every 3 months to obtain self-reported CVD outcomes.

The primary outcome of this analysis was incident dysglycemia and subsequent composite outcomes (including incident CVD events or all-cause death). Dysglycemia was including diabetes or prediabetes. Diabetes was defined by the FPG ≥ 126 mg/dL (7.0 mmol/L), participant self-report of diabetes at annual examination or use of diabetic medication. Prediabetes was defined by the American Diabetes Association criteria, as impaired fasting glucose with FPG 100–125 mg/dL (5.6–6.9 mmol/L).

**Statistical Analysis**

Descriptive statistics summarized baseline characteristics, presenting mean ± standard deviation (SD) for normally distributed continuous variables, median with interquartile range (IQR) for non-normally distributed variables, and proportions were reported for categorical variables. All participants with normoglycemia at baseline, randomly assigned, were included in the analysis based on the intention-to-treat principle.

For participants with normoglycemia at baseline, cumulative incidence and 3-year absolute risk analyses for glucometabolic and nonfatal CVD outcomes were conducted using a competing risk proportional hazards regression model. Non-CVD death was treated as a competing risk for CVD outcomes, with all-cause death treated as a competing risk for incident dysglycemia. For participants with new-onset dysglycemia, cumulative incidence at and 2-year absolute risk analyses CVD outcomes or were conducted using a Cox proportional hazards regression model.

The proportional hazards assumption for treatment effects was assessed by examining Schoenfeld residuals, and no evidence of significant departures were found. Interactions between treatment effect and prespecified subgroups (age (<75 vs. ≥75 years), sex, and race (black vs. non-black) were assessed with a likelihood-ratio test for the interaction with the use of Hommel-adjusted P values. All analyses were performed using R, version 4.3.1 (R Foundation for Statistical Computing, Vienna, Austria). All P values were two sided, and P values < 0.05 were considered to indicate statistical significance.

**Table S1. Baseline characteristics of SPRINT participants with normoglycemia by treatment group.**

| **Variables** | **Overall**  **(n=5027)** | **Standard**  **(n=2505)** | **Intensive**  **(n=2522)** |
| --- | --- | --- | --- |
| Mean age, years | 68.01 ± 9.59 | 68.01 ± 9.65 | 68.01 ± 9.54 |
| Male, n (%) | 3027 (60.21) | 1534 (61.24) | 1493 (59.20) |
| Race/ethnicity, n (%) |  |  |  |
| Black**^*^** | 1691 (33.64) | 861 (34.37) | 830 (32.91) |
| Non-black | 3147 (62.60) | 1552 (61.96) | 1595 (63.24) |
| Mean SBP (SD), mmHg | 140.24 (15.70) | 140.16 (15.48) | 140.33 (15.91) |
| Mean DBP (SD), mmHg | 78.56 (11.89) | 78.51 (11.89) | 78.61 (11.90) |
| Mean heart rate (SD), bpm | 65.62 (11.34) | 65.61 (11.51) | 65.63 (11.17) |
| Mean BMI (SD), kg/m^2^ | 29.82 (5.76) | 29.79 (5.76) | 29.84 (5.76) |
| Smoking status, n (%) |  |  |  |
| Never | 2291 (45.63) | 1138(45.50) | 1153(45.75) |
| Previous | 2015 (40.13) | 1023(40.90) | 992(39.37) |
| Current | 715 (14.24) | 340(13.59) | 375(14.88) |
| Mean glucose (SD), mg/dL | 90.66 (6.39) | 90.76 (6.31) | 90.56 (6.46) |
| Mean triglycerides (SD), mg/mL | 116.02 (71.73) | 117.70 (76.77) | 114.36 (66.33) |
| Mean total cholesterol (SD), mg/mL | 191.65 (41.21) | 190.62 (40.22) | 192.67 (42.15) |
| Mean HDL-C (SD), mg/mL | 54.88 (15.04) | 54.60 (15.09) | 55.16 (14.98) |
| Mean LDL-C (SD), mg/mL | 113.71 (35.15) | 112.63 (34.03) | 114.77 (36.19) |
| Mean serum creatinine(SD), mg/dL | 1.07 (0.35) | 1.08 (0.36) | 1.07 (0.35) |
| Mean eGFR, mL/min/1.73 m^2^ | 71.47 (20.85) | 71.45 (20.93) | 71.49 (20.78) |
| Mean ASCVD 10 years risk (SD) | 19.57 (10.72) | 19.58 (10.64) | 19.57 (10.80) |
| Comorbidities, n (%) |  |  |  |
| History of clinical CVD | 790 (15.72) | 398 (15.89) | 392 (15.54) |
| History of subclinical CVD | 271 (5.39) | 398 (15.89) | 392 (15.54) |
| CKD | 1447 (28.78) | 719 (28.70) | 728 (28.87) |
| Aspirin use, n (%) | 2630 (52.39) | 1291 (51.64) | 1339 (53.13) |
| Statin use, n (%) | 1916 (38.11) | 976 (38.96) | 940 (37.27) |
| Beta-blocker use, n (%) | 1810 (36.01) | 880(35.13) | 930(36.88) |

SPRINT, Systolic Blood Pressure Intervention Trial; SD, standard deviation; SBP, systolic blood pressure; DBP, diastolic blood pressure; BMI, body mass index; HDL-C, high-density lipoprotein cholesterol; LDL-C, low-density lipoprotein cholesterol; eGFR, estimated glomerular filtration rate; ASCVD, atherosclerotic cardiovascular disease; CVD, cardiovascular disease; CKD, chronic kidney disease.

**^*^**Black race includes participants identifying as Hispanic Black and/or Black as part of multiracial identification, consistent with the SPRINT trial's subgroup analysis.
